# Perceived Coercion Among Patients Admitted in Psychiatric Wards: Italian Results of the EUNOMIA Study

**DOI:** 10.3389/fpsyt.2019.00316

**Published:** 2019-05-21

**Authors:** Gaia Sampogna, Mario Luciano, Valeria Del Vecchio, Benedetta Pocai, Carmela Palummo, Giovanna Fico, Vincenzo Giallonardo, Corrado De Rosa, Andrea Fiorillo

**Affiliations:** Department of Psychiatry, University of Campania “Luigi Vanvitelli”, Napoli, Italy

**Keywords:** perceived coercion, involuntary admission, formal coercion, Cantril Ladder, severe mental disorder

## Abstract

The decision to use coercive measures (restraint, seclusion and forced medication) in psychiatric practice is controversial in mental health care. The EUNOMIA study was funded by the European Commission and carried out in 11 countries in order to develop European recommendations for good clinical practice on the use of coercive measures. The aim of the study is to identify sociodemographic and clinical predictors of the levels of perceived coercion in a sample of Italian patients with severe mental disorders at hospital admission.

A total of 294 patients were recruited in five Italian psychiatric hospitals and screened with the MacArthur Perceived Coercion Scale to explore the levels of perceived coercion. Patients were assessed three times: within the first seven days after admission as well as after 1 and 3 months. At each time point, data on changes of perceived coercion, assessed by the Cantril Ladder of Perceived Coercion Scale, information on coercive measures received during hospitalization and the levels of satisfaction with the received treatments were collected.

According to the multivariable regression model, being compulsorily admitted (OR: 2.5; 95% CI: 1.3–3.3, p < .000), being male (OR: 0.7; 95% CI: 0.9-1.4; p < .01), being older (OR: 0.03; 95% CI: 0.01–0.06) and less satisfied with received treatments (OR: -0.2; 95% CI: -0.3 to -0.1; p < .05) are all associated with higher levels of perceived coercion, even after controlling for the use of any coercive measure during hospitalization.

Satisfaction with received treatment predicts the levels of perceived coercion and this should represent an important challenge for mental health professionals.

## Introduction

Formal coercion is defined as coercion exercised within the regulations of a given mental health legislation (1). In the framework of formal coercion, different types of coercive measures are included, namely involuntary admission, forced pharmacological treatments, use of physical restraint, and isolation (2, 3).

The use of coercive measures represents a controversial and highly debated issue in mental health. Adopting formal coercion could be necessary to provide treatments to patients with a poor level of insight, those not able to seek for psychiatric help and those who cannot receive the needed treatments (4). However, it has been pointed out that coercive treatments are less effective than voluntary ones and can lead to patients’ distrust, reduced satisfaction with received treatments and decreased level of engagement with mental health services (5, 6).

Patients are increasingly recognized as key decision makers in mental health care (7). Allowing patients to choose treatments and have a say in their care could be associated with a better outcome and increased medication adherence (8, 9). Therefore, it is important to evaluate and assess patients’ subjective experience of feeling coerced, defined as “perceived coercion” (2, 10).

Little is known about the impact of perceived coercion on clinical outcomes after discharge. Katsakou et al. (11) found that satisfaction with treatment among involuntary patients was associated with high levels of perceived coercion during admission and treatment, rather than with the documented extent of coercive measures. Priebe et al. (12) documented that patients’ views on treatment within the first week are a relevant indicator for the long-term prognosis of involuntarily admitted patients. The formal legal admission status and the use of coercive measures are often not directly associated with the subjective experience of being coerced (13, 10), and perceived coercion is a more accurate measure of coercion (14). Several studies have showed that patients’ subjective experience of coercion in hospital is mostly related to the perceived “negative pressure” at admission (i.e., use of threats and of force), to the feeling of not being involved in decisions regarding admission, and to the feeling of being treated with no respect and no consideration (4, 15, 16). However, Gardner et al. (17) have highlighted that the levels of perceived coercion at admission tend to be stable over time, even when patients’ opinions about the actual need of admission improve. Available data on determinants of patients’ perceived coercion at admission in psychiatric wards are conflicting.

Not surprisingly, several studies (18, 19, 20) have shown that involuntarily admitted patients perceive higher levels of coercion compared to those voluntarily admitted. However, perceived coercion is only partially related to the formal status of admission, and it is confounded by several socio-demographic and clinical variables, including age, ethnicity (perceived coercion is higher in non-white populations), diagnosis, insight of the illness and severity of symptoms (10, 21, 22). To our knowledge, no study has been carried out on perceived coercion in Italy, the country with the longest experience of community mental health care. This paper aims to 1) identify the sociodemographic and clinical characteristics associated with high levels of perceived coercion at admission in psychiatric wards and 2) assess the relationship between the levels of perceived coercion at admission and the levels of satisfaction with received care after three months of hospitalization in a sample of Italian patients with severe mental disorders.

## Materials and Methods

Data reported in this study have been collected within the “European evaluation of coercion in psychiatry and harmonization of best clinical practice (EUNOMIA) project,” funded by the European Commission and carried out in 11 European countries (Bulgaria, Czech Republic, Germany, Greece, Italy, Lithuania, Poland, Slovakia, Spain, Sweden and UK). The characteristics of participating mental health facilities, aims, and methods of the whole study have been reported in detail elsewhere (3, 23). For the purposes of this manuscript, we included data on patients recruited in five Italian psychiatric wards (Naples, Salerno, Nocera Inferiore, Sant’Angelo dei Lombardi, Polla).

In order to be eligible for the study, patients should have high levels of subjective experience of feeling coerced (perceived coercion) at admission. The patients’ subjective experience of being coerced at enrollment was assessed with the Mac Arthur Scale for Perceived Coercion (24). Patients with a score >3 from the MacArthur Perceived Coercion Scale were recruited. All instruments, including the MacArthur Scale, have been translated and back-translated before recruitment. Patients affected by dementia, alcohol or drug acute intoxication, eating disorders requiring forced nutrition or severe cognitive impairment were excluded from the study. All enrolled patients received adequate information on the study’s aims and provided written informed consent to participate in the study.

Patients were assessed three times: within the first seven days after admission (T0), after one month (T1) and after three months (T2). Sociodemographic characteristics have been collected with an ad-hoc schedule. Diagnoses were recorded at discharge according to the ICD-10 criteria and have been grouped into the following: 1) schizophrenia and other psychotic disorders (F20-F29); 2) affective disorders (F30-F39); and 3) other disorders. Patients’ global functioning was assessed with the Global Assessment of Functioning (GAF, 25) and the severity of psychiatric symptoms with the Brief Psychiatric Rating Scale (BPRS), 24-item version (26).

At baseline, the levels of perceived coercion have been evaluated using the “perceived coercion” items from the MacArthur Admission Experience Survey (patient interview version), while at T1 and T2 the levels of perceived coercion and pressures concerning hospital admission have been evaluated using the Cantril Ladder of Perceived Coercion and items from the Nordic Study on Coercion (patient interview version) (27–29). The Cantril Ladder is rated on a 10-point Likert scale, from 1 (minimum level of perceived coercion) to 10 (maximum level of perceived coercion).

At T2, the Client’s Assessment of Treatment (CAT) and an ad-hoc schedule on the use of mental health services after discharge and on patients’ opinions regarding the decision of the index hospitalization (e.g., “who decided in favor of hospitalization?”) were used (29). The CAT evaluates patients’ satisfaction with treatment during the previous three months. It consists of seven items, exploring satisfaction with received treatments, with the treating clinician and with other mental health professionals, with medications, with other received treatments, and the level of satisfaction with the global received care. Each item is rated on a 10-point Likert scale, from 0 (“not at all”) to 10 (“yes entirely”).

As part of the assessment procedure, an ad-hoc schedule was used to collect information on coercive measures. According to the study protocol (29), coercive measures were defined as follows: seclusion is the involuntary placement of an individual alone in a locked room; restraint is the fixation of at least one of the patient’s limbs by a mechanical device or at least one limb being held by staff for longer than 15 minutes; forced medication refers to activities which use restraint or high psychological pressure to administer medication against the patient’s will; involuntary detention is defined by any of the following criteria: a) the patient is initially admitted on a legally voluntary basis and withdraws his consent to hospitalization at a later stage; b) the legally defined time period (different between countries) in which the hospital is allowed to initially detain a patient without applying for a decision of the responsible legal authorities has passed; or c) the detention is based on the authorization of legal authorities.

All other details regarding the study protocol have been published elsewhere (29).

## Ethical Standards

The authors assert that all procedures contributing to this work comply with the ethical standards of the relevant national and institutional committees on human experimentation and with the Helsinki Declaration of 1975, as revised in 2008.

## Statistical Analyses

The socio-demographic and clinical characteristics of voluntary and involuntary admitted patients were compared using chi-square or t-test for independent samples, as appropriate. Pearson’s rho was used to evaluate correlations between the levels of perceived coercion and the levels of satisfaction with treatments.

In order to identify predictors of the levels of perceived coercion, a linear regression model has been performed using the score at the Cantril Ladder as the main outcome. Before performing the regression model, normal distribution was checked and confirmed. Therefore, a linear regression model was developed, entering in the model several socio-demographic and clinical variables identified from previous studies in the field.

Statistical analyses were performed using the Statistical Package for Social Sciences (SPSS), version 17.0. For all analyses, the level of statistical significance was set at p < .05.

## Results

### Sample Description

The global sample consists of 294 patients, whose main sociodemographic and clinical characteristics are shown in **Table 1**. The majority of patients (N = 165; 56%) were voluntarily admitted and suffered from psychosis (62.7%), with a prevalence of positive and manic symptoms at BPRS. Most of the patients had been previously admitted in psychiatric wards (78.6%) (**Table 1**). Compulsorily admitted patients were more frequently male (p < .001), employed (p < .01), with higher levels of positive and manic/hostility symptoms (p < .000) and lower depression/anxiety symptoms (p < .001) (**Table 1**).

**Table 1:** Socio-demographic and clinical characteristics of the sample (N = 294).

	Total sample (n = 294)	Voluntarily admitted patients (N = 165)	Compulsorily admitted patients (n = 129)	p-value
Gender, male, % (N)	52.7 (155)	55.8 (92)	34.9 (45)	.001
Age, years, M (± sd)	39.9 (10.5)	40.5 (10.7)	39 (10.3)	NS
Married, yes, % (N)	24.8 (73)	27.3 (45)	21.7 (28=	NS
Diagnosis, % (N)				
Schizophrenia and other psychotic disorders Affective disorders Other	62.7 (183) 23.3 (68) 14 (41)	59.4 (98) 26.1 (43) 14.5 (24)	66.9 (85) 19.7 (25) 13.4 (17)	NS NS NS
Employed, yes, % (N)	16.6 (48)	11.7 (20)	24.6 (31)	.01
Years of education, M (± sd)	15.9 (3.8)	15.7 (4)	16.1 (3.5)	NS
Previous hospitalizations, yes, % (N)	78.6 (231)	83.6 (138)	72.1 (93)	NS
Previous compulsory hospitalizations, yes, % (N)	70.0 (204)	35.1 (57)	57.4 (77)	.000
BPRS subscales (score range: 1-7)				
Positive symptoms, M (± sd)	3.2 (1.3)	2.9 (1.3)	3.7 (1.2)	.000
Negative symptoms, M (± sd)	2.6 (1.1)	2.5 (1.1)	2.7 (1.2)	NS
Manic/excitement symptoms, M (± sd)	3.7 (1.4)	2.1 (1.2)	3.3 (1.5)	.000
Depression/anxiety symptoms, M (± sd)	2.5 (1.1)	2.7 (1.1)	2.3 (0.9)	.001
GAF (score range: 0-100), M (± sd)	43.3 (15.1)	43.7 (15.3)	42.7 (15.0)	NS
CAT global score (score range: 0-10), M (± sd)	6.7 (2.0)	7.5 (1.5)	5.7 (2.1)	.000
Cantril Ladder (score range: 0-10), M (± sd)	7.3 (2.4)	6.4 (2.5)	8.5 (1.6)	.000

N, number; BPRS, Brief Psychiatric Rating Scale; GAF, Global Assessment of Functioning; CAT, Clients’ Scale for Assessment of Treatment; NS, Not Significant; M, mean; sd, standard deviation.

At baseline, patients reported a considerably high level of perceived coercion, with a mean score of 7.3 ± 2.4 at the Cantril Ladder of Perceived Coercion scale. Regarding patients’ perceived coercion and pressure at admission, 20% of voluntary admitted patients reported that the decision for admission was made by other people. In particular, they attributed the decision to be hospitalized to other people (69%), mainly close relatives (80%), mental health professionals (16%), police officers (2.5%), friends or colleagues (1.5%). Only 16% of these patients spontaneously decided to be admitted, while 15% affirmed that the decision of hospitalization was made together with other people.

Involuntarily admitted patients reported that the decision for hospitalization was made by close relatives (63%), mental health professionals (18%), police officers (18%), friends or colleagues (1%). On the other hand, 9% of compulsorily admitted patients did not report high levels of perceived coercion, confirming having spontaneously decided to be hospitalized.

At admission, patients were quite satisfied with received treatments and recognized that the hospitalization was helpful; moreover, they reported that they felt respected and regarded well during the hospitalization. The correlation analyses confirmed that patients reporting higher levels of satisfaction at T2 were those reporting lower levels of coercion at admission (rho = -.193, p < .01). Furthermore, we found an improvement in the levels of satisfaction with received treatments over time (**Table 2**).

**Table 2:** Levels of treatment satisfaction and correlations with the levels of perceived coercion.

Score 0-10 at CAT items	Changes in levels of treatments satisfaction			Correlations between levels of treatments satisfaction and perceived coercion at hospital admission
	Baseline	T1	T2	
Do you believe you are receiving the right treatment/care for you here?	6.6 (1.9)	7.4 (1.5)	7.7 (1.2)	-.279*
Does your therapist/case manager/keyworker understand you and is he/she engaged in your treatment/care?	6.6 (2.1)	7.5 (1.8)	7.9 (1.2)	-.291*
Are relations with other staff members here pleasant or unpleasant for you?	7.0 (2.1)	7.7 (1.8)	8.1 (1.3)	-.222*
Do you believe you are receiving the right medication for you?	6.6 (2.2)	7.4 (1.7)	7.9 (1.3)	-.247*
Do you believe the other elements of treatment/care here are right for you?	6.6 (2.7)	7.6 (1.8)	7.9 (1.3)	-.213**
Do you feel respected and regarded well here?	7.1 (2.2)	7.8 (1.8)	8.1 (1.4)	-.178**
Has treatment/care here been being helpful for you?	6.8 (2.3)	7.6 (1.7)	8.0 (1.3)	-.193**

*p-value < .000; **p-value < .01.

CAT, Client's Assessment of Treatment.

### Coercive Measures

Regardless of the legal status at admission, 84 patients (28.6%) reported to have received one or more coercive measures during hospitalization: 66 (22.4%) patients received forced medication, 26 (8.8%) patients were physically restrained, and 20 (6.8%) patients were isolated from other patients. Patients who received at least one coercive measure during the hospitalization were more frequently male (p < .01), with higher levels of positive (p < .000), negative (p < .001), and manic-excitement (p < .000) symptoms, and with lower levels of depression/anxiety subscales at BPRS subscales (p < .01). Moreover, they reported higher levels of perceived coercion (p < .000) and worse global functioning at GAF scores (p < .01) compared to those not receiving coercive measures (**Table 3**).

**Table 3:** Socio-demographic and clinical characteristics differences according to the use of coercive measures (regardless the formal admission status).

	Use of coercive measures		
	Yes (N = 84)	No (N = 210)	p-value
Gender, male, % (N)	67.9 (57)	46.7 (98)	.01
Age, years, M (± sd)	39.4 (10.9)	40.1 (10.4)	NS
BPRS subscales (score range: 1-7)			
Positive symptoms, M (± sd)	3.5 (1.4)	2.3 (1.3)	.000
Negative symptoms, M (± sd)	2.9 (1.1)	2.4 (1.1)	.001
Manic/excitement symptoms, M (± sd)	3.8 (1.1)	3.0 (1.4)	.000
Depression/anxiety symptoms, M (± sd)	2.2 (0.9)	2.6 (1.1)	.01
GAF (score range: 0-100), M (± sd)	39.2 (13.0)	44.9 (15.6)	.01
Cantril Ladder (score range: 0-10), M (± sd)	6.9 (1.9)	7.7 (1.4)	.000

N, number; BPRS, Brief Psychiatric Rating Scale; GAF, Global Assessment of Functioning; NS, Not Significant; M, mean; sd, standard deviation

### Predictors of the Levels of Perceived Coercion at Admission

According to the multivariable regression model, several predictors of perceived coercion were identified. In particular, the levels of perceived coercion are higher in patients being compulsorily admitted (OR: 2.5; 95% CI: 1.3–3.3; p < .000), male (OR: 0.7; 95% CI: 0.9-1.4; p < .01), older (OR: 0.03; 95% CI: 0.01-0.06; p < .05) and less satisfied with received treatments (OR: -0.2; 95% CI: -0.3 to -0.1; p < .05), even after controlling for the use of coercive measures during hospitalization (**Table 4**).

**Table 4:** Predictors of levels of perceived coercion at the admission.

Number of subjects included in the analysis	294			
F (df)	9.619 (14)			
P	.000			
Adjusted R square	0.310			
Constant	10.1 (7.7 to 12.6)			
	OR	95% CIs		p-value
Gender, ref. category female	0.8	0.9	1.4	.01
Previous psychiatric hospitalizations, yes	0.4	-0.7	0.9	NS
Employed, yes	0.8	-0.1	1.5	NS
Legal status at admission, involuntary admission	2.5	1.3	3.3	.000
Receiving any coercive measures during admission, yes	-0.21	-1	0.6	NS
Diagnosis, ref. category “Other”				
- Schizophrenia and other psychotic disorders	-0.4	-1.3	0.5	NS
- Affective disorders	-0.6	-1.6	0.4	NS
Age	0.03	0.04	0.06	.05
BPRS subscale, positive symptoms	0.2	-0.1	0.6	NS
BPRS subscale, negative symptoms	0.3	-0.6	0.1	NS
BPRS subscale, manic/excitement symptoms	-0.1	-0.3	-0.3	NS
BPRS subscale, depression/anxiety symptoms	-0.4	-0.7	-0.1	NS
GAF global score	-0.1	-1.4	-0.1	.01
CAT global score	-0.2	-0.3	-0.1	.05

BPRS, Brief Psychiatric Rating Scale; GAF, Global assessment of Functioning; F-test; df, degree of freedom; OR, Odds Ratio; CIs, Confidence Intervals.

## Discussion

This study, carried out as part of the collaborative European multicenter project EUNOMIA, represents the first effort to describe the levels of perceived coercion in a sample of Italian patients, using a robust and reliable methodology. In particular, several standardized assessment tools for evaluating formal and perceived coercion have been administered by trained mental health professionals, who were already engaged in the clinical activities of the participating mental health facilities.

According to our study, the levels of perceived coercion are related to the legal status at admission and to several patients’ sociodemographic and clinical characteristics, such as age, gender and previous admissions. Involuntarily admitted patients showed higher levels of coercion compared to the voluntarily admitted ones, recognizing that the decision of admission to the psychiatric ward was made by other people. These data are consistent with previous studies (2, 10, 30, 31) and suggest that patients tend to have similar experiences during involuntary admission—regardless of national legislations. This confirms the idea that compulsory admission, as well as the adoption of any other coercive intervention in psychiatric settings, should be considered as the last acceptable treatment option and should be adopted only when all other therapeutic interventions fail (4, 32–34). Involuntary admission, although being used to manage patients during the acute phases of their disorder, could lead to high levels of perceived coercion and to high levels of skepticism from patients toward the efficacy of provided interventions. One possible way to reduce the negative impact of involuntary admissions on patients’ perceived coercion can be the implementation in routine settings of the Joint Crisis Plans (JCPs) and the Patients’ Advanced Directives (PADs) (35). These plans are based on patients’ anticipated will about possible treatments to be received during acute crises. Despite the fact that these strategies seem to be promising, their implementation in routine care is still poor.

Another relevant finding is that male patients tend to feel more coerced than females, being also more frequently involuntarily admitted. This finding is in line with those found in other studies carried out in different socio-cultural contexts (34, 36).

Regarding the decision about hospital admission, more than half of the patients reported that this choice was made by relatives on their behalf. This finding emphasizes the need to involve patients’ relatives already in the initial phases of treatment, providing them adequate information on patients’ disorders, teaching them strategies for the detection of early warning signs and correctly managing the situation, in order to reduce the risk of compulsory admissions (37–39).

Furthermore, we found that the level of perceived coercion is influenced by the level of functioning, but not by the severity of clinical symptomatology. A possible explanation could be that worse psychosocial functioning is often associated with a poor level of insight, which can be an obstacle to being motivated for treatment and the acceptance of hospitalization. This aspect is controversial, since previous studies (40) have suggested a close link between clinical status and levels of perceived coercion, while other studies did not (22, 30). However, in our sample we found that patients with higher levels of personal functioning reported low levels of perceived coercion, while no differences were found comparing the formal status at admission and the levels of personal and social functioning.

In our sample, patients with high levels of perceived coercion reported low satisfaction with treatment and process of care. Improving patients’ satisfaction with received treatments represents a challenge for mental health professionals. Our findings are in line with available literature and suggest that reducing patients’ feelings of coercion might lead to higher overall satisfaction (11, 41).

Patients’ satisfaction with treatment seems to be more strongly linked to perceived coercion rather than to formal coercion, since perceived coercion is largely based on the overall experience of involuntary treatments and on modalities of treatment negotiation with patients. Good empathy, realistic and explicit communication would allow patients to feel more involved in decisions regarding their health (42), to improve patient-clinician relationship (43) and to promote patients’ recovery (44, 45). In particular, a shared approach in decision-making should be adopted in order to improve not only patients’ satisfaction with received treatments, but also patients’ adherence to treatments (46–49).

The study has some limitations that must be acknowledged. First, the study was conducted within the framework of the EUNOMIA project and data were collected in the period 2003-2005. Inpatient bed coverage in Italy is lower compared to other European countries participating in the EUNOMIA study sites (e.g., Naples: 4,7 per 100 000; Wroclaw: 30,6 per 100.000; Dresden: 63,7). Therefore, it may be that in Italy we recruited a highly selected (and severely symptomatic and functionally impaired) inpatient population compared to the other European countries.

Furthermore, the methodological choice of including patients with high levels of perceived coercion (i.e., MacArthur scale score >3) may have selected the sample and limited the interpretation of a complex phenomenon such as that of perceived coercion. Since the evaluation of perceived coercion is mainly based on patient-reported questionnaires, recall bias, memory-loss and lack of knowledge on definitions of coercive measures may limit the generalizability of the findings, although the adopted instruments are reliable and have been previously validated.

Another limitation is that the participating mental health centers are all located in Southern Italy, whereas several organizational differences exist throughout Italy. Therefore, a study involving different centers from different Italian regions may be advisable for an in-depth understanding of this phenomenon in Italy.

## Ethics Statement

The study has been approved by ethical committees of each participating centre. Data of patients reported in the paper refer only to those recruited in the Naples centre.

## Author Contributions

GS, ML, VDV and AF contributed to the conception and design of the work and in drafting the manuscript; BP, CP, GF, VG and CDR contributed to the collection, analysis and interpretation of data. GS, ML, CDR and AF developed the statistical analysis plan and drafted the manuscript. All authors provided the approval for publication of the manuscript.

## Funding

This work was supported by a grant from the European Union’s Seventh Framework Programme (Grant agreement number: QLG4-CT-2002-01036).
